# Treatment strategies and outcomes in patients with non-ST-elevation acute coronary syndrome in the Netherlands

**DOI:** 10.1007/s12471-018-1218-x

**Published:** 2018-12-14

**Authors:** P. Damman, J. J. Piek

**Affiliations:** 1 Department of Cardiology, Radboud University Medical Center, Nijmegen, The Netherlands; 2 Department of Cardiology, Academic Medical Centre, Location Meibergdreef, University of Amsterdam, Amsterdam, The Netherlands

Acute coronary syndrome (ACS) is predominantly caused by acute luminal narrowing due to sudden thrombus formation or plaque haemorrhage imposed on an atherosclerotic plaque [1]. A luminal thrombus forms as a direct consequence of plaque rupture or erosion. The initial electrocardiogram can be used to differentiate ST-elevation myocardial infarction (STEMI) and non-ST-elevation acute coronary syndrome (NSTE-ACS). Patients with STEMI are characterised by a complete and persistent occlusion of a large epicardial coronary artery and are best managed with immediate revascularisation by primary percutaneous coronary intervention [2]. Primary percutaneous coronary intervention (PCI) for STEMI is associated with a reduction in mortality compared with other reperfusion strategies [3].

In NSTE-ACS, typically an angiographically significant stenosis is observed while epicardial flow is maintained. Therefore, multiple treatment strategies with regard to coronary angiography and revascularisation have been investigated over the years. The first step is whether to proceed with a routine invasive or selective invasive strategy. A routine invasive strategy consists of angiography within 24 to 72 hours, while a selective invasive strategy consists of coronary angiography only in cases of refractory angina and/or inducible ischaemia by non-invasive stress testing. The Dutch multicentre ICTUS (Invasive versus Conservative Treatment in Unstable coronary Syndromes) trial has demonstrated that both treatment strategies resulted in comparable long-term death or myocardial infarction [4]. A patient-pooled meta-analysis of three trials showed that long-term rates of cardiovascular death or myocardial infarction were lower with routine invasive strategy, and that the largest absolute effect was seen in higher-risk patients [5]. The difference between the discrepant results can probably be explained by the high angiography and revascularisation rates in the ICTUS trial. Current European guidelines recommend a routine invasive strategy for patients with intermediate-to-high-risk characteristics, while the selective invasive strategy is reserved for low-risk patients [6].

The second decision involves the optimal timing of angiography after pursuing the routine invasive approach. Different timings are defined as immediate, early invasive strategy (within 24 hrs), or delayed invasive strategy (after 24 hrs). Several trials have investigated the immediate, or STEMI-like approach which was not associated with improved outcomes [7–9]. A recent meta-analysis concluded that compared with a delayed invasive strategy an early invasive strategy does not reduce mortality in all patients with NSTE-ACS [10]. However, an early invasive strategy might reduce mortality in high-risk patients characterised by a high Global Registry of Acute Coronary Events (GRACE) risk score (above 140). For this reason, European guidelines recommend angiography within 24 hours for high-risk patients, including transfer to a PCI centre [6]. Based on multiple considerations, including the availability of catheterisation laboratories in the majority of Dutch cardiology departments where coronary angiography is routinely performed in NSTE-ACS patients, the Dutch ACS working group does not consider referral within 24 hours as a prerequisite [11].

In the current issue of the Netherlands Heart Journal, Badings et al. describe the use, timing and outcome of coronary angiography in patients with high-risk NSTE-ACS in daily clinical practice in the Netherlands [12]. Consecutive ACS patients who presented at the Isala hospital in Zwolle are described. In Zwolle, consecutive patients were enrolled in the BAMI (Dutch abbreviation for ‘Treatment of Acute Myocardial Ischaemia’) registry between 2006 and 2014. The main findings were as follows: 1) The use of coronary angiography increased (from 77 to 90%); 2) There was a significant decrease in median time to coronary angiography; 3) There was an increase in patients undergoing early invasive strategy; 4) A higher GRACE risk score was associated with a delayed invasive strategy; 5) No difference was observed in mortality, reinfarction and bleeding event at 30-day follow-up between early and delayed invasive strategy after adjustment for confounding factors.

The increased use of coronary angiography, decrease in the median time to coronary angiography and increase of patients undergoing early invasive strategy can probably be explained by the ESC guideline recommendations. This observation is in line with a similar report from the Swedish SWEDEHEART registry [13]. However, contrary to what might be expected based on these recommendations, patients with a higher baseline risk profile as indicated by the GRACE risk score less frequently underwent an early invasive strategy. This inverse relationship between risk profile and the use of invasive treatment, the treatment-risk paradox, has been described previously [13, 14]. The explanation for this treatment-paradox is likely multifactorial [14]. Treating physicians may fail to recognise high-risk clinical features or to properly integrate them into overall risk stratification. Moreover, physicians may be hesitant to recommend or perform invasive procedures for patients at high risk for treatment complications. ‘Therapeutic nihilism’ (belief that treatment might be ineffective in patients with poor outcome) may also play a role. To be noted, factors that are not routinely captured in risk scores or studies, such as functional status, patient’s preference and frailty, do influence treatment decisions in everyday practice.

Finally, even though higher-risk patients were treated with a delayed invasive strategy, no difference was observed in clinical outcomes between an early invasive strategy and a delayed invasive strategy. A subgroup analysis showed that this finding was consistent in patients with a GRACE score above 140. Although these results have to be interpreted in the light of the limitations of a non-randomised comparison, the results are in line with previous Dutch randomised studies regarding timing of coronary angiography/intervention in NSTE-ACS patients [15, 16]. In addition to the above-mentioned availability of catheterisation laboratories in the majority of Dutch cardiology departments, this important finding might be explained by the correct identification of true high-risk patients by the treating physician. In the recently published Danish VERDICT trial, NSTE-ACS patients were randomised to coronary angiography within 12 hours or within 48 to 72 hours [17]. Although no differences in outcomes were observed, there was a benefit of coronary angiography within 12 hours with regard to long-term outcomes in patients with a GRACE risk score above 140. The question then arises whether high-risk NSTE-ACS patients presenting to a cardiology department without coronary angiography capabilities should be transferred to a PCI-capable centre. We believe that a Dutch randomised trial investigating these treatment strategies in high-risk patients is needed, because the clinical and logistical (ambulance and catheterisation facilities/laboratories) implications of inter-facility transfer are significant.
